# Association between left atrial volume index and infarct volume in patients with ischemic stroke

**DOI:** 10.3389/fneur.2023.1265037

**Published:** 2023-11-20

**Authors:** Moayad Homssi, Venkatesh Balaji, Cenai Zhang, James Shin, Ajay Gupta, Hooman Kamel

**Affiliations:** ^1^Department of Radiology, Weill Cornell Medicine, New York, NY, United States; ^2^Clinical and Translational Neuroscience Unit, Feil Family Brain and Mind Research Institute and Department of Neurology, Weill Cornell Medicine, New York, NY, United States

**Keywords:** radiology, stroke, left atrial enlargement, infarct, infarct volume

## Abstract

**Background:**

Left atrial volume index (LAVI) is one marker of atrial myopathy, which is increasingly being recognized as a cause of cardioembolic stroke even in the absence of atrial fibrillation. Cardiac embolism is associated with larger strokes than other stroke mechanisms. The purpose of this study was to examine the association between LAVI and total brain infarct volume in patients with ischemic stroke.

**Methods:**

This was a retrospective study of 545 patients prospectively enrolled in the Cornell ActuE Stroke Academic Registry (CAESAR), which includes all acute ischemic stroke patients admitted to our hospital since 2011. LAVI measurements were obtained from our echocardiography image store system (Xclera, Philips Healthcare). Brain infarcts on diffusion-weighted images (DWI) were manually segmented and infarct volume was obtained on 3D Slicer. We used multiple linear regression models adjusted for age, sex, race, and vascular comorbidities including atrial fibrillation.

**Results:**

Among 2,945 CAESAR patients, 545 patients had both total infarct volume and LAVI measured. We found an association between LAVI and log-transformed total brain infarct volume in both unadjusted (β = 0.018; *p* = 0.002) and adjusted (β = 0.024; *p* = 0.001) models.

**Conclusion:**

We found that larger left atrial volume was associated with larger brain infarcts. This association was independent of known cardioembolic risk factors such as atrial fibrillation and heart failure. These findings support the concept that atrial myopathy may be a source of cardiac embolism even in the absence of traditionally recognized mechanisms such as atrial fibrillation.

## Introduction

Stroke is one of the leading causes of morbidity and mortality worldwide (1). Occult cardiac embolism is believed to be a common underlying source of strokes that are currently of unknown mechanism, otherwise known as cryptogenic stroke or embolic stroke of undetermined source (ESUS). While patients with atrial fibrillation have long been known to have an increased risk of stroke, atrial myopathy in the absence of atrial fibrillation is being recognized as a potential cause of cardiac embolism and stroke (2). Left atrial volume index (LAVI) is one marker of atrial myopathy and has previously been shown to be associated with stroke risk. The importance of LAVI as a biomarker lies in its ability to predict development atrial fibrillation, which is particularly important in ESUS patients.

Cardioembolic stroke is associated with larger brain infarct volume (3). Given that atrial myopathy is associated with cardiac embolism, and cardiac embolism tends to lead to larger brain infarcts, we hypothesized that LAVI would be associated with larger brain infarct volume among patients with ischemic stroke.

## Methods

### Design

This was a cross-sectional study using data from the Cornell AcutE Stroke Academic Registry (CAESAR). The study was approved by Weill Cornell Medical College's institutional review board, and the requirement for informed consent was waived. Deidentified data and the analytic methods that produced the results of this study are available upon reasonable request from the corresponding author.

### Patient population

All patients hospitalized at New York-Presbyterian Hospital/Weill Cornell Medical Center for acute stroke are prospectively enrolled in the American Heart Association's Get With The Guidelines (GWTG)—Stroke registry. Trained hospital analysts prospectively collect data on demographics, vascular risk factors and comorbidities, stroke severity, and in-hospital treatments and outcomes. CAESAR combines the GWTG data plus additional retrospectively collected clinical, laboratory, and radiographic data. All cases of ischemic stroke are reviewed by a panel of at least three neurologists who adjudicate the etiology per the Trial of Org 10172 in Acute Stroke Treatment (TOAST) classification and per the consensus definition for embolic stroke of undetermined source (ESUS). For this analysis, we included all ischemic stroke patients who were registered in CAESAR from 2011 through 2016 except those with a subtype of other known cause, who represented a small and heterogeneous fraction of our sample. For this study we included patients who had both infarct volume and LAVI measurements.

### Measurements

The exposure variable was the left atrial volume index (LAVI) measured during transthoracic echocardiography (TTE). LAVI values were imported directly from our hospital's echocardiographic image management system (Xcelera, Philips Healthcare) into a Microsoft SQL server and merged into the main CAESAR registry. We excluded TTEs without a recorded LAVI measurement. For patients with multiple TTEs with LAVI measurements, we included the qualifying study performed closest in time to the index stroke. LAVI measurements were based on automated measurements taken at the time of echocardiography, and board-certified cardiologist with specific training in echocardiography verified LAVI measurements <10 or >125 mL/m, thresholds that were chosen based on clinical expertise and prior to beginning analysis.

**Figure 1:**
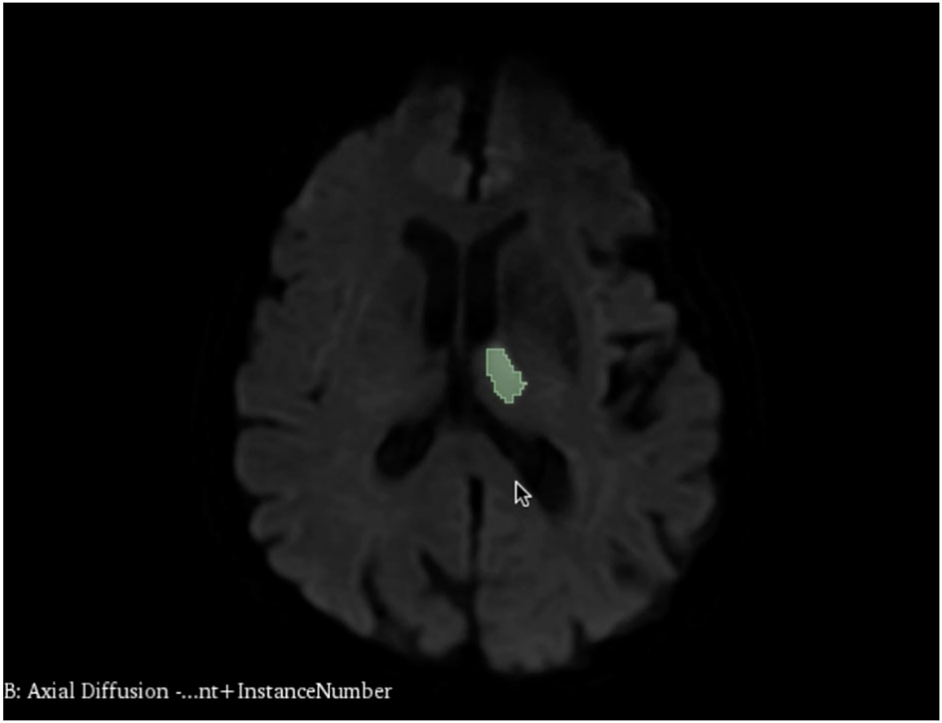
Segmented brain infarct in the left thalamus.

Brain infarcts on diffusion-weighted images (DWI) were manually segmented and infarct volume was generated on 3D Slicer. Two medical students with prior knowledge in neuroanatomy and who received dedicated training on DWI interpretation independently loaded DWI images from our DICOM database into Slicer. Each image was carefully examined for the presence of DWI hyperintensities (Figure 1). Temporal lobe and cerebellar diffusion artifacts were carefully avoided during the segmentation process. Allocation of territory of infarct was based on the blood supply region of the infarcted brain parenchyma, classified as right anterior, left anterior, or posterior circulation. DWI hyperintensities in each territory were segmented individually in cases of bilateral anterior circulation infarctions and those involving both the anterior and posterior circulations.

Global thresholding and level tracing functions on Slicer were used to segment DWI hyperintensities. In cases of large multifocal DWI hyperintensities, the image interpreters used global thresholding to manually set a threshold that included the hyperintense region of interest without including unrelated hyperintensities in the brain including artifact. The segmentation was then carefully and conservatively fine-tuned using various default tools available on Slicer such as erase and draw functions. In cases with small, well-circumscribed DWI hyperintensities, the interpreters used the level tracing tool, which allowed the interpreters to select a region of hyperintense voxels consistent with infarcted tissue and automatically found a closed path of voxels with the same intensity value as the voxel of origin, thereby segmenting uniformly intense regions in each slice.

### Statistical analysis

We used descriptive statistics to characterize the patient cohorts. For descriptive purposes, we categorized LAVI at mean value. Left atrial enlargement was defined as LAVI ≥ 34 mL/m^2^, per established echocardiography guidelines (4). Infarct volume was natural logarithmic transformed to normalize the data. Associations between infarct volume and LAVI as continuous measures were examined by linear regression and adjusted for demographics and vascular comorbidities. Model covariates were age, sex, race, atrial fibrillation, heart failure, diabetes, hypertension, coronary artery disease, and valvular disease. All the covariates were pre-specified and included in the model regardless of significance. Sensitivity analysis was performed where we additionally adjusted for left ventricular ejection fraction. Analyses were performed using Stata/MP, version 15.1 (Stata Corp, TX). The threshold of statistical significance was set at 0.05.

## Results

Among 2,945 CAESAR patients, 1,524 had available LAVI measurements, and of these, 545 patients (18.5%) also had total infarct volume. Among these patients, 68 had stroke from large-artery atherosclerosis, 154 from cardioembolism, 54 from small-vessel occlusion, and 31 from another cause, whereas 238 had cryptogenic stroke (Table 1). The mean LAVI was 36.1 [standard deviation (SD), 16.2] mL/m^2^ and the median infarct volume was 1.9 [interquartile range (IQR), 0.4–10.7] mm^3^. The established threshold for moderately abnormal left atrial size is 34 mL/m^2^.

**Table 1:** Characteristics of patients in the CAESAR registry included in analysis of LAVI and brain infarct volume.

	**Group ≤ 34**	**Group > 34 (LA enlargement)**
	**N** = **271**	**N** = **274**
Age, mean (SD), yrs	65.3 (14.9)	74.1 (12.9)
Female	101 (54.0)	99 (51.3)
**Race—includes missing**		
White	49 (18.1)	44 (16.1)
Black	12 (4.4)	13 (4.7)
Asian	7 (2.6)	14 (5.1)
Other	15 (5.5)	13 (4.7)
Missing	188 (69.4)	190 (69.3)
**Insurance status**		
Commercial	104 (38.4)	96 (35.0)
Medicare	82 (30.3)	112 (40.9)
Medicaid	26 (9.6)	26 (9.5)
Other	59 (21.8)	40 (14.6)
Atrial fibrillation, *n* (%)	14 (5.2)	74 (27.0)
Coronary artery disease *n* (%)	36 (13.3)	56 (20.4)
Heart failure *n* (%)	5 (1.9)	26 (9.5)
Diabetes *n* (%)	76 (28.0)	67 (24.5)
Hypertension *n* (%)	175 (64.6)	215 (78.5)
Prior stroke *n* (%)	44 (16.2)	53 (19.3)
Chronic kidney disease *n* (%)	13 (4.8)	24 (8.8)
Valvular disease *n* (%)	2 (0.7)	5 (1.8)
Active tobacco use *n* (%)	21 (7.8)	16 (5.8)
Onset-to-arrival time, median (IQR), hours	10.9 (2.6–38.3)	6.8 (2.3–24.4)
Arrived by ambulance *n* (%)	104 (38.4)	124 (45.3)
IV TPA administered *n* (%)	24 (8.9)	26 (9.5)
Door-to-needle time, median (IQR), minutes	48 (31–68)	32.5 (28.5–57)
Reference NIHSS score, median (IQR)	3 (1–6)	4 (1–11)
**TOAST ischemic stroke subtype** ***n*** **(%)**		
Large-artery atherosclerosis	46 (17.0)	22 (8.0)
Cardioembolic	33 (12.2)	121 (44.2)
Small-vessel occlusion	37 (13.7)	17 (6.2)
Other	21 (7.8)	10 (3.7)
Cryptogenic	114 (42.1)	73 (26.6)
Incomplete work-up	3 (1.1)	13 (4.7)
Multiple causes	17 (6.3)	18 (6.6)
**Ambulatory status at discharge**		
Not walking	13 (4.9)	25 (9.4)
Walking with assistance	52 (19.4)	56 (21.1)
Walking independently	203 (75.8)	185 (69.6)

Table 2 examines median infarct volumes subclassified by TOAST ischemic stroke subtype. Generally, cardioembolic strokes are associated with large infarct volumes while small-vessel lacunar strokes are seen to be associated with much smaller infarcts. In large-artery atherosclerosis, the infarct volume was 2.0 mm^3^ with an interquartile range (IQR) of 0.5–6.6 mm^3^. In cardioembolic stroke, the infarct volume of 4.7 mm^3^ (IQR: 0.9–18.8 mm^3^). In small-vessel occlusion, the infarct volume was measured at 0.3 mm^3^ (IQR of 0.2–0.6 mm^3^). In cryptogenic stroke, the volume was 1.5 mm^3^ (IQR: 0.5–9.4 mm^3^). The category labeled “Other” displayed an infarct volume of 1.8 mm^3^ (IQR of 0.4–9.1 mm^3^). In strokes with an incomplete work-up, the volume was determined to be 1.6 mm^3^ (IQR: 0.4–6.8 mm^3^). Finally, strokes attributed to multiple causes showed an infarct volume of 0.7 mm^3^ (IQR: 0.3–3.8 mm^3^).

**Table 2:** Infarct volume by TOAST ischemic stroke subtype.

**TOAST ischemic stroke subtype**	**Total infarct volume (mm^3^), median (IQR)**
Large-artery atherosclerosis	2.0 (0.5–6.6)
Cardioembolic stroke	4.7 (0.9–18.8)
Small-vessel occlusion	0.3 (0.2–0.6)
Cryptogenic stroke	1.5 (0.5–9.4)
Other	1.8 (0.4–9.1)
Incomplete work-up	1.6 (0.4–6.8)
Multiple causes	0.7 (0.3–3.8)

Cardioembolic strokes are associated with larger infarct volumes while small-vessel occlusions are associated with much smaller infarcts.

To meet the linear regression assumptions, we log transformed total infarct volume. In the univariate linear regression model, we observed a significant association between larger brain infarct volume and LAVI, with a 1.8% increase in total brain infarct volume per each mL/m^2^ increase in LAVI (*p* = 0.002, Table 3). This association remained significant after controlling for covariates mentioned above. After adjusting for demographic and comorbidities, we found a 2.1% increase in total brain infarct volume per mL/m^2^ increase in LAVI (*p* = 0.006, Table 3).

**Table 3:** Regression models relating LAVI to infarct volume.

	***N***	**β**	***P*-Value**
Model 1	545	0.018	0.002
Model 2	380	0.024	0.001
Model 3	380	0.021	0.006
Sensitivity analysis	286	0.016	0.077

Model 1: unadjusted univariate linear regression model; Model 2: multivariate regression model adjusted for age, gender, race; Model 3: multivariate regression model additionally adjusted for atrial fibrillation, heart failure, diabetes, hypertension, coronary artery disease, and valvular disease; In the sensitivity analysis, we additionally adjusted for left ventricular ejection fraction.

We conducted a sensitivity analysis by further adjusting for left ventricular ejection fraction. The result showed that after this additional adjustment, there was no significant association between total brain infarct volume and LAVI (*p* = 0.077, Table 3).

## Discussion

Our study demonstrated a correlation between left atrial volume and brain infarct volume among patients with stroke. There are sparse data on the relationship between atrial cardiopathy and infarct volume in this population. A recently published study demonstrates a strong association between cardioembolic stroke etiology and larger brain infarct volume (3). Additionally, a previous study established a link between left atrial volume and the presence of cardioembolic stroke (5). Multiple studies have found associations between various markers of atrial cardiopathy, including left atrial size and function, and the risk of stroke, vascular brain injury, and dementia (2–6).

LAVI's utility as a biomarker is of particular interest in patients with ESUS. One recent study has demonstrated that the proportion of patients with atrial cardiopathy is significantly higher among ESUS patients, compared to those with large artery atherosclerosis or small-vessel occlusion. This study also found that ESUS patients with atrial cardiopathy had similar baseline characteristics to those with a cardioembolic source of stroke and have a higher risk of death compared to those without atrial cardiopathy (7). A recent meta-analysis (8) attempted to analyze the relationship between LAVI (rather than LA diameter) and stroke risk, but concluded the available data was limited and controversial. Our study will contribute to the existing literature in this capacity.

Other recently published articles provide supporting context that structural causes of atrial cardiopathy (i.e., fibrosis, atrial remodeling) may precede and contribute to the pathogenesis of atrial fibrillation. It has additionally been seen that LAVI was independently associated with detection of atrial fibrillation in ESUS patients who then underwent outpatient cardiac event monitoring (9). Thus, atrial cardiopathy may result in embolic stroke prior to the development of atrial fibrillation, elucidating a potential cause of cryptogenic stroke (10). Measures of left atrial enlargement, such as LAVI, hold promise in diagnosing atrial cardiopathy and may guide secondary prevention of stroke with initiation of DOAC therapy. In fact, measures of left atrial enlargement have been seen to significantly improve both 2-year and 5-year stroke risk prediction when used in conjunction with the CHA2DS2-VASc score (11). Consistent with the previously discussed studies, this increase in risk was particularly notable among patients with no documented history of atrial fibrillation.

In this context, our study provides novel findings suggesting a link between left atrial volume and brain infarct volume independent of other known vascular comorbidities. These findings suggest that atrial cardiopathy, independent of atrial fibrillation, may lead to cardiac embolism which is associated with more severe stroke and larger brain infarct volume. These findings require confirmation in future studies better adjusting for potential confounders. Ultimately, these findings may support further efforts to delineate optimal measures of atrial cardiopathy and test preventive strategies such as anticoagulation or left atrial appendage occlusion in patients at high risk.

There are several limitations to our study. First, the cross-sectional design may result in bias, most notably reverse causation, in which patients with larger brain infarct volume subsequently experienced left atrial dilation, potentially through neurocardiogenic mechanisms. Second, there may be residual confounding from shared factors, such as frailty, which may explain both larger left atrial volume and larger brain infarct volumes. Third, atrial fibrillation is strongly associated with atrial cardiomyopathy and resultant LA enlargement. The prevalence of atrial fibrillation in our cohort is 32.2%, with 27% prevalence in the LA enlargement group. Future studies ought to investigate the effect of LA enlargement in patients with current sinus rhythm as such homogenous patient cohort will provide a clearer insight into the role of LA enlargement on stroke, independent of the confounding effects of atrial fibrillation (12). Fourth, left atrial volume may not be an ideal or comprehensive measure of atrial cardiopathy, and more a more refined exposure variable may have led to more reliable estimates of the association. Fifth, the observational nature of our study lacks the ability to definitively establish a causative link between LAVI and infarct volume. While a correlation was found, this does not necessarily prove that patients with increased LAVI exhibit larger brain infarct volumes. Sixth, the retrospective nature of our study introduces some limitations to our study as retrospective studies in general include recall bias and lack of control over specific data collection, including important covariates such as obstructive sleep apnea and large-vessel occlusion status. Lastly, our sample size was relatively small and limited to patients from a single center, which limits the generalizability of our results.

In summary, we found an association between left atrial volume and brain infarct volume, independent of atrial fibrillation, in stroke patients. These findings are consistent with the hypothesis that atrial cardiopathy may be associated with cardiac embolism independent of atrial fibrillation.

## Data availability statement

The raw data supporting the conclusions of this article will be made available by the authors, without undue reservation.

## Ethics statement

The studies involving humans were approved by Weill Cornell Medicine Institutional Review Board. The studies were conducted in accordance with the local legislation and institutional requirements. Written informed consent for participation was not required from the participants or the participants' legal guardians/next of kin in accordance with the national legislation and institutional requirements.

## Author contributions

MH: Conceptualization, Investigation, Writing—original draft. VB: Conceptualization, Investigation, Writing—original draft. CZ: Data curation, Formal analysis, Writing—review & editing. JS: Conceptualization, Supervision, Writing—review & editing. AG: Conceptualization, Supervision, Writing—review & editing. HK: Conceptualization, Supervision, Writing—review & editing.
